# Optimal phytase supplementation levels for broilers differ by thermal condition for phosphorus utilization but not for growth or bone mineralization

**DOI:** 10.1016/j.psj.2026.107415

**Published:** 2026-07-08

**Authors:** Hansol Kim, Sunday A. Adedokun

**Affiliations:** Department of Animal and Food Sciences, University of Kentucky, Lexington, KY, 40546, USA

**Keywords:** Broiler, Heat stress, Optimal level, Phosphorus utilization, Phytase

## Abstract

This study evaluated the effects of reducing dietary calcium (**Ca**) and phosphorus (**P**) concentrations and supplementing phytase on broilers reared under thermoneutral or cyclic heat stress (**HS**) conditions, and estimated the optimal phytase level for each environment. A total of 420 one-d-old male broiler chicks were assigned to a 2 × 5 factorial arrangement consisting of 2 rearing temperatures and 5 diets: a positive control (**PC**), a Ca- and P-deficient negative control (**NC**), and the NC supplemented with 500, 1,000, or 1,500 phytase units (**FTU**) per kilogram of diet. Birds were reared under identical temperatures from d 0 to 7 and then exposed to either thermoneutral or cyclic HS conditions from d 7 to 21. Growth performance, bone mineralization, and nutrient digestibility and utilization were measured, and one-slope broken-line analysis was used to estimate optimal phytase levels. Phytase supplementation linearly or quadratically increased (*P* < 0.05) ADG, ADFI, bone mineralization, and Ca and P utilization. Estimated optimal phytase levels for ADG, ADFI, tibia breaking strength, and nitrogen excretion did not differ between thermoneutral and HS conditions, were generally within the range of approximately 500 to 800 FTU/kg, and produced plateau responses that did not differ from the least squares means of broilers fed the PC diet under either environment. In contrast, the optimal phytase level for P utilization was greater (*P* = 0.029) under HS than under thermoneutral conditions (987 vs. 536 FTU/kg), and supplementing the NC diet with these estimated optimal phytase levels resulted in greater (*P* < 0.001) P utilization than in broilers fed the PC diet. In conclusion, phytase supplementation improved broiler performance, bone mineralization, and P utilization in NC diets under both environments, although the optimal inclusion level depended on the response criterion and environmental condition, indicating that supplementing an NC diet with the optimal level of phytase can restore most responses to the level of the PC diet and can even result in greater P utilization than the PC diet.

## Introduction

Phosphorus (**P**) and calcium (**Ca**) are essential minerals in broiler nutrition because they are required for skeletal development and numerous metabolic processes, including energy metabolism, cellular signaling, and acid-base balance (Adedokun and Adeola, 2013). However, a large proportion of P in corn-soybean meal-based diets is present as phytate, which is poorly utilized by poultry because of limited endogenous phytase activity in the gastrointestinal tract (Adejumo et al., 2021). Phytate can also form complexes with Ca and other nutrients, thereby reducing nutrient digestibility and utilization (Lei et al., 2013). Therefore, exogenous phytase is widely used in broiler diets to hydrolyze phytate, improve P digestibility and utilization, reduce reliance on inorganic P sources, and decrease P excretion (Adeola et al., 2004; Olayiwola and Adedokun, 2025). Previous studies have shown that phytase supplementation in Ca- and P-reduced diets can improve growth performance, bone mineralization, and nutrient digestibility, and may allow partial or complete replacement of inorganic phosphate sources without compromising productive responses (Adejumo et al., 2021; Marchal et al., 2021).

Heat stress (**HS**) is another major factor affecting broiler production (Olayiwola and Adedokun, 2025). Broilers exposed to high ambient temperature commonly reduce feed intake, which can decrease growth performance and alter nutrient supply relative to physiological requirements (Teyssier et al., 2023). In commercial production, birds are often exposed to cyclic rather than constant HS, with high temperatures during part of the day and partial relief during cooler periods (Andretta et al., 2021). Previous studies have shown that cyclic HS can reduce ADG and ADFI, although the magnitude of impairment may be less severe than that observed under continuous HS (de Souza et al., 2016; Teyssier et al., 2022). Heat stress can also alter intestinal function, nutrient digestibility, blood biochemistry, inflammatory responses, and mineral metabolism, suggesting that its effects are not limited to reduced feed intake alone (Andretta et al., 2021; Teyssier et al., 2022).

Because HS reduces feed intake and alters nutrient metabolism, the response to phytase under HS may differ from that under thermoneutral (**TN**) conditions. A lower feed intake under HS may reduce the absolute intake of digestible Ca and P, increasing the bird’s dependence on phytase-mediated release of phytate-bound P. In addition, P homeostasis is tightly regulated through endocrine, renal, skeletal, and intestinal mechanisms. When P supply is limited, birds may increase P conservation and absorption through mechanisms involving calcitriol synthesis, renal P reabsorption, and altered intestinal phosphate transport (Proszkowiec-Weglarz and Angel, 2013; Omotoso et al., 2023). Therefore, the optimal phytase level under HS may not be identical to that under TN conditions, particularly for P utilization responses.

Although phytase has been extensively studied in broilers fed nutrient-reduced diets (Letourneau-Montminy et al., 2008; Akter et al., 2016; Sommerfeld et al., 2018), information is limited on whether the phytase level required to optimize growth performance, bone mineralization, and P utilization differs between TN and cyclic HS conditions. In addition, the phytase level required to maximize growth performance may not be the same as that required to maximize bone mineralization or P utilization. This distinction is important because phytase is used not only to support growth, but also to improve skeletal integrity, reduce inorganic P use, and decrease environmental P losses. Another practical consideration is phytase stability during exposure to elevated temperature, because loss of enzyme activity during storage may confound biological responses to phytase under hot conditions (Sulabo et al., 2011; De Jong et al., 2016, 2017).

Therefore, the objectives of the present study were to determine the effects of reducing dietary Ca and P and supplementing graded levels of phytase on growth performance, bone mineralization, and nutrient digestibility and utilization in broilers reared under TN or cyclic HS conditions; estimate the optimal phytase level for selected response criteria within each thermal environment; compare the estimated plateau responses of broilers fed the negative control (**NC**) diet supplemented with phytase with the responses of birds fed the positive control (**PC**) diet; and evaluate the effect of environmental exposure on analyzed phytase activity. It was hypothesized that cyclic HS would reduce growth performance and increase the severity of nutrient limitation in broilers fed the NC diet, that phytase supplementation would improve growth performance, bone mineralization, and nutrient utilization under both TN and HS conditions, and that the phytase level required to maximize P utilization would be greater under HS than under TN conditions. It was also hypothesized that prolonged exposure to HS conditions would accelerate the decline in analyzed phytase activity during storage.

## Materials and methods

The experimental procedures and management of birds were approved by the Institutional Animal Care and Use Committee of the University of Kentucky (Approval No.: 2022-4157 V.8).

### Birds, experimental diets, and experimental design

A total of 420 one-d-old male Cobb 500 by-product broiler breeder chicks (initial BW = 41.9 ± 1.0 g) were randomly assigned to a 2 × 5 factorial arrangement consisting of 2 rearing temperatures and 5 dietary treatments in a completely randomized design. Each treatment had 6 replicate cages with 7 birds per cage. The 2 rearing temperatures were TN and cyclic HS. Five corn-soybean meal-based diets were formulated to meet or exceed nutrient requirement estimates recommended in the Cobb 500 Broiler Nutrition Guide (Cobb-Vantress, 2022), except for Ca and P (Table 1). The dietary treatments were as follows: (1) positive control (**PC**), that met the recommended Ca and P levels; (2) negative control (**NC**), containing 83% and 55% of recommended Ca and non-phytate P levels in the PC diet, respectively; (3-5) the NC diet supplemented with 500, 1,000, or 1,500 FTU/kg of phytase. The NC diet and phytase-supplemented diets were produced from a single basal diet and differed only by the addition of a bacterial (*Aspergillus niger*) granulated hybrid 6-phytase (Natuphos® E 5000 G; BASF Corporation, Florham Park, NJ, USA). The product contained 5,000 phytase units (**FTU**) per gram, and was included at 0.1, 0.2, or 0.3 g/kg of diet to achieve the target levels. One FTU was defined as the amount of enzyme that liberates 1 μmol of inorganic P per minute from 0.0051mol/L sodium phytate at pH 5.5 and 37°C (Adeola et al., 2004). All diets were provided in mash form and contained 0.5% titanium dioxide as an indigestible marker for digestibility and utilization calculations. Birds were housed in battery cages (0.61 × 0.51 × 0.36 m; Model SG12; Alternative Design Manufacturing, Siloam Springs, AR, USA) with ad libitum access to feed and water throughout the experiment. From d 0 to 7, room temperature was maintained identically in both TN and HS rooms (28-30°C). From d 7 to 21, the HS room was subjected to cyclic HS (34-38°C for 8 h/d from 08:00 to 16:00, followed by 26-29°C from 16:00 to 08:00), whereas the TN room was maintained at 26-29°C. Relative humidity was maintained similarly across all treatments throughout the experimental period (33.8 ± 2.5%). Room temperature and humidity were monitored using 2 thermometers (HOBO ZW data loggers, Onset Computer Corp., Bourne, MA, USA) positioned at different locations within each room. To avoid potential reductions in phytase activity caused by exposure to elevated temperatures, all diets were stored under TN conditions and provided to the feeders on a daily basis. A lighting schedule of 22L:2D was applied throughout the study.

**Table 1:** Ingredient and chemical composition of experimental diets (as-fed basis)^1^.

Item, g/kg	Positive control	Negative control^2^
Ingredient		
Ground corn	551.5	568.1
Soybean meal, 47% CP	368.0	365.0
Soybean oil	36.0	30.0
Dicalcium phosphate	16.3	5.3
Limestone	9.4	12.5
Sodium chloride	4.8	4.9
_DL_-Met, 99.0%	3.9	4.0
_L_-Lys·HCl, 78.8%	1.6	1.6
_L_-Thr, 99.0%	0.8	0.8
_L_-Val, 99.0%	0.5	0.5
Vitamin-mineral premix^3^	2.3	2.3
Titanium dioxide	5.0	5.0
Calculated values		
AME, kcal/kg	3,086	3,086
CP	221.0	221.0
Phytate phosphorus	2.6	2.6
Non-phytate phosphorus	4.6	2.5
Analyzed values		
Gross energy, kcal/kg	4,120	4,138
CP	223.4	222.2
Calcium	11.4	10.0
Phosphorus	9.4	6.4
Phytase activity, FTU/kg	< 70	< 70

1 The PC diet contained 100% of the recommended calcium and phosphorus levels, whereas the NC diet contained 83% and 71% of the recommended levels for calcium and phosphorus, respectively.

2 Three additional diets were formulated by supplementing 3 levels of phytase (Natuphos® E 5000 G, BASF Corporation, Florham Park, NJ) into the negative control diet. The phytase product contained 5,000 phytase unit (FTU) per gram of phytase, and 0.1, 0.2, or 0.3 g of phytase/kg of diet were added to achieve 500, 1,000, or 1,500 FTU/kg, respectively.

3 The vitamin-mineral premix provided the following quantities per kilogram of complete diet: vitamin A (retinyl acetate), 8,820 IU; vitamin D_3_ (cholecalciferol), 2,822 IU; vitamin E (_DL_-α-tocopheryl acetate), 26 IU; vitamin K_3_ (menadione), 0.73 mg; thiamine, 1.76 mg; riboflavin, 6.17 mg; niacin, 44 mg; _d_-pantothenic acid (_D_-calcium pantothenate), 14 mg; pyridoxine, 4 mg; biotin, 0.18 mg; folic acid, 0.88 mg; choline (choline chloride), 383 mg; vitamin B_12_ (cyanocobalamin), 0.02 mg; iron (ferrous sulfate), 32 mg; manganese (manganese sulfate), 51 mg; copper (cupric sulfate), 8 mg; zinc (zinc oxide), 60 mg; iodine (potassium iodide), 1.48 mg; and selenium (sodium selenite), 0.24 mg.

### Evaluation of phytase stability and sample collection

Representative samples of each diet were collected at the feed mill immediately after mixing and prior to the initiation of the experiment. To evaluate the effect of prolonged exposure to room temperature on phytase activity, 2 kg of each diet were placed in separate feeders identical to those used for birds and stored in both TN and HS rooms for 21 d (5 feeders per room). Diets in each of the 5 feeders in each room were manually mixed twice daily to simulate feeding conditions. On d 21, the diet in each of the 5 feeders per room was emptied into a prelabelled clean plastic container. The diet was thoroughly mixed and subsampled. This step was repeated for each room. These samples as well as those collected at the feed mill were sent to the same lab for phytase activities.

Growth performance variables, including ADG, ADFI, and gain-to-feed ratio (**G:F**), were determined for d 0-7, 7-14, 14-21, and 0-21 after adjusting for mortality (Kim et al., 2025). From d 20 to 21, excreta samples were collected from each cage and immediately stored at −20°C. On d 21, one bird per cage with BW closest to the cage mean was euthanized via argon asphyxiation, and the right tibia and femur were collected from 2 birds per cage and stored at −20°C for bone analyses. For ileal digestibility determination, all 7 birds per cage were euthanized, and ileal digesta samples were collected from the entire ileum by flushing with distilled water. Digesta from birds within each cage were pooled, homogenized, and stored at −20°C until freeze-drying.

### Chemical and bone analyses

Excreta samples were dried at 55°C in a forced-air oven for 5 d to constant weight. Ileal digesta samples were lyophilized for 5 d prior to analysis. Diets, excreta, and ileal digesta were ground (< 0.5 mm) using a centrifugal mill (ZM 200; Retsch GmbH, Haan, Germany). Phytase activity of stored diets was analyzed to determine the effect of environmental exposure (method 2000.12; AOAC, 2006). Dry matter (method 934.01), nitrogen (method 990.03), calcium (method 975.03B(b)), and phosphorus (method 966.01) were analyzed according to AOAC (2006) at the Agricultural Experiment Station Chemical Laboratories (University of Missouri, Columbia, MO, USA). Gross energy was determined using a bomb calorimeter (Model 6200; Parr Instruments, Moline, IL, USA), calibrated with benzoic acid. Titanium concentrations were determined according to Myers et al. (2004).

Bones were cleaned of adhering tissues and subjected to bone breaking strength (BBS) determination using an Instron testing machine (Model 4301; Instron Corp., Canton, MA, USA) at a loading rate of 50 mm/min. Bones were then dried at 105°C for 24 h, defatted using petroleum ether extraction, air-dried under the hood at room temperature for about 8 h and placed in the oven at 105°C overnight, weighed, and ashed overnight at 600°C (Bauer et al., 2025). Bone ash percentage was calculated on a fat-free dry basis.

### Calculations

For digestibility and utilization of energy and nutrient calculations, chemical analyses from diet samples collected immediately after mixing at the feed mill prior to environmental exposure were used. Apparent ileal digestibility (**AID**) and apparent utilization of gross energy and nutrients were calculated using the index method (Adejumo et al., 2021) as:

AID or apparent utilization (%) = 100 × [1 – (Nutr_out_/Nutr_in_) × (Ti_in_/Ti_out_)],

where Nutr_out_ and Nutr_in_ represent the concentrations of energy (kcal/kg) or nutrients (%) in the ileal digesta or excreta and in the experimental diets, respectively. Ti_in_ and Ti_out_ represent the titanium concentrations (%) in the diet and in the ileal digesta or excreta, respectively. Nitrogen-corrected AME was calculated according to Hill and Anderson (1958):

AMEn (kcal/kg) = AME (kcal/kg) – N retention (g/kg) × 8.22 kcal/g of retained N.

Nitrogen intake, retention, and excretion were calculated as described by Njeri et al. (2026).

### Statistical analyses

To evaluate the effect of room temperature on phytase activity, regression analyses were conducted separately for TN and HS conditions. For diets without phytase supplementation (PC and NC; 0 FTU/kg), analyzed phytase activity was below the detection limit (< 70 FTU/kg) and therefore assigned a value of zero. Regression lines were forced through the origin using the REG procedure of SAS.

Calculated phytase supplementation levels were used in the statistical analyses. Normality was assessed using the UNIVARIATE procedure of SAS, and homogeneity of variance was evaluated using Levene’s test via the GLM procedure. Both a priori and post hoc power analyses were conducted assuming a Type I error rate of 0.05 and statistical power of 0.80. The minimum required number of replicates per treatment was estimated to be 4.63, therefore, 6 replicates per treatment were considered adequate. Outliers were assessed using the interquartile range method (Kim et al., 2022a), and none were identified.

The original design consisted of a 2 × 5 factorial arrangement and the dataset was partitioned into two analytical subsets. The first subset was analyzed as a 2 × 2 factorial to evaluate the effects of rearing temperature (TN vs. HS) and dietary Ca and P reduction (PC vs. NC). The second subset was analyzed as a 2 × 4 factorial to evaluate rearing temperature and graded phytase supplementation (0, 500, 1,000, and 1,500 FTU/kg). The NC treatment under both environmental conditions was included in both analytical subsets. Data were analyzed using the MIXED procedure of SAS (SAS Inst. Inc., Cary, NC, USA). Rearing temperature, dietary treatment, and their interaction were included as fixed effects. For the 2 × 2 analysis, orthogonal contrasts were used to evaluate main effects and interactions. Least squares means (**LSM**) were estimated for each treatment combination, and when interactions were significant, means were separated using Tukey’s adjustment for multiple comparisons. For the 2 × 4 analysis, polynomial orthogonal contrasts were applied to determine linear and quadratic responses to phytase supplementation and their interactions with temperature. Contrast coefficients were generated using the IML procedure of SAS. Because cyclic HS was imposed only from d 7 onward, all birds were reared under TN conditions during d 0 to 7. Therefore, growth performance data for this period were analyzed separately from later periods. For the 2 × 2 subset, only dietary treatment (PC vs. NC) was included in the model. For the 2 × 4 subset, only the linear and quadratic effects of phytase supplementation were evaluated.

Within each environmental condition, the optimal phytase level, the corresponding plateau response, and their SE were estimated using a one-slope broken-line regression model fitted with the NLIN procedure of SAS (Kim et al., 2022b). For each response variable, the estimated optimal phytase levels between TN and HS conditions were compared using a two-sample z-test. In addition, within each environmental condition, the LSM response of broilers fed the PC diet was compared with the estimated plateau response obtained from the one-slope broken-line analysis using a two-sample z-test. The experimental unit was a cage, and statistical significance was declared at *P* < 0.05.

## Results

**Figure 1:**
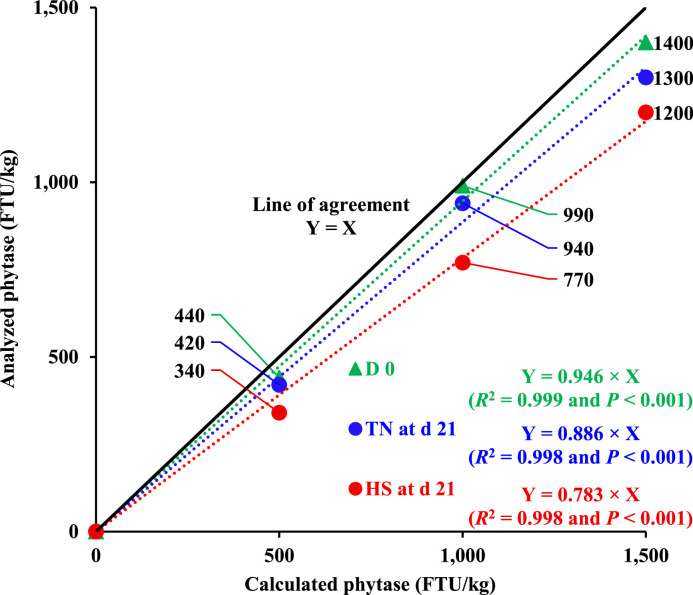
Effects of storage duration and temperature on analyzed phytase activity in broiler diets. Diets containing 0, 500, 1,000, or 1,500 phytase units (FTU) per kg of diet were sampled immediately after mixing at the feed mill (d 0) and analyzed for phytase activity. To evaluate the effects of storage temperature, 2 kg of each diet were placed in feeders identical to those used for birds and stored in thermoneutral (TN) or cyclic heat stress (HS) rooms for 21 d. Diets were manually mixed twice daily to simulate feeding conditions. On d 21, feed samples were collected from each feeder, composited within room, subsampled, and analyzed for phytase activity. From d 0 to 7, temperature was identical in both TN and HS rooms (28–30°C). From d 7 to 21, the HS room was subjected to cyclic heat stress (34–38°C for 8 h/d from 08:00 to 16:00, followed by 26–29°C for the remaining 16 h), whereas the TN room was maintained at 26–29°C. For diets without phytase supplementation (0 FTU/kg), analyzed phytase activity was below the detection limit (< 70 FTU/kg) and was therefore assigned a value of zero. Regression lines were forced through the origin.

Analyzed phytase activity values at d 0 of storage were within the expected analytical variation for phytase activity (Figure 1). At d 0 of storage, analyzed phytase activity decreased by 5.4% relative to calculated values (*R*^2^ = 0.999, *P* < 0.001). After 21 d of storage, analyzed phytase activity decreased by 6.3% under TN (*R*^2^ = 0.998, *P* < 0.001) and by 17.2% under HS (*R*^2^ = 0.998, *P* < 0.001) relative to d 0 of storage. This represents an additional 11.6% reduction under HS compared with TN.

Birds fed the PC diet showed greater ADG, ADFI, and BW at d 7 compared with those fed the NC diet (*P* < 0.01; Table 2). Over the overall experimental period (d 0-21), birds reared under TN conditions exhibited greater (*P* = 0.034) ADG than those under HS. Birds fed the PC diet also showed greater (*P* < 0.01) ADG and G:F compared with birds fed the NC diet. An interaction between temperature and diet was observed for ADFI during d 0-21 (*P* = 0.048), such that HS did not affect ADFI in birds fed the PC diet but reduced ADFI in birds fed the NC diet compared with TN.

**Table 2:** Effects of temperature (T) and reduced dietary calcium and phosphorus concentrations on growth performance in broilers.

Item	T^1^:	TN		HS		SEM	*P*-values		
	Diet^2^:	PC	NC	PC	NC		T	Diet	T × Diet
*n*		6	6	6	6	-	-	-	-
d 0 to 7									
Initial BW, g/bird		42.3	42.0	42.0	41.4	0.3	-	0.201	-
ADG, g/bird/d		19.6	17.7	19.6	17.8	0.6	-	0.008	-
ADFI, g/bird/d		25.6	23.8	24.7	23.0	0.4	-	< 0.001	-
G:F, g/g		0.765	0.743	0.791	0.775	0.023	-	0.412	-
Final BW, g/bird		179.2	165.0	179.8	166.0	4.4	-	0.005	-
d 7 to 14									
ADG, g/bird/d		41.1	35.0	41.4	31.0	1.3	0.173	< 0.001	0.123
ADFI, g/bird/d		51.9	42.9	48.2	39.1	1.6	0.032	< 0.001	0.988
G:F, g/g		0.798^b^	0.816^ab^	0.858^a^	0.791^b^	0.018	0.339	0.194	0.030
Final BW, g/bird		467.0	409.8	469.4	383.2	11.1	0.287	< 0.001	0.204
d 14 to 21									
ADG, g/bird/d		70.4	65.1	69.0	56.2	2.0	0.020	< 0.001	0.080
ADFI, g/bird/d		84.9^a^	84.4^a^	86.0^a^	75.5^b^	2.2	0.087	0.020	0.033
G:F, g/g		0.832	0.772	0.803	0.744	0.019	0.142	0.006	0.973
Final BW, g/bird		960.0	865.5	952.6	776.8	21.2	0.035	< 0.001	0.069
d 0 to 21									
ADG, g/bird/d		43.7	39.3	43.3	35.0	1.0	0.034	< 0.001	0.073
ADFI, g/bird/d		54.2^a^	50.3^b^	53.0^ab^	45.9^c^	0.8	0.002	< 0.001	0.048
G:F, g/g		0.808	0.780	0.818	0.762	0.013	0.773	0.005	0.303

G:F = gain-to-feed ratio; HS = heat stress; NC = negative control; PC = positive control; TN = thermoneutral.

^a-c^Means within a row without a common superscript letter differ (*P* < 0.05) based on the interaction.

1 Temperatures were identical between the TN and HS rooms from d 0 to 7 (28-30°C). From d 7 to 21, the HS room temperature was increased to 34-38°C for 8 h/d (08:00 to 16:00) and then reduced to 26-29°C from 16:00 to 08:00 the following day, representing a cyclic heat stress pattern that was maintained until the end of the study. The TN room temperature was maintained at 26-29°C from d 7 to 21.

2 The PC diet contained 100% of the recommended calcium and phosphorus levels, whereas the NC diet contained 83% and 71% of the recommended levels for calcium and phosphorus, respectively.

From d 0 to 7, increasing phytase supplementation resulted in linear and quadratic increases in ADG, ADFI, and final BW (*P* < 0.05; Table 3). During d 0-21, phytase supplementation linearly increased (*P* < 0.001) G:F. An interaction between temperature and the linear effect of phytase supplementation was observed for ADG and ADFI (*P* < 0.05), such that both variables increased linearly with increasing phytase levels under TN and HS conditions, but the rate of increase was greater under HS, being approximately 2.5-fold higher for ADG and 2.0-fold higher for ADFI compared with TN.

**Table 3:** Effects of temperature (T) and phytase supplementation levels on growth performance in broilers.

Item	T^1^:	TN				HS				RMSE	*P*-values for contrasts^2^				
	Phytase (FTU/kg):	0	500	1,000	1,500	0	500	1,000	1,500		T	L	Q	T × L	T × Q
*n*		6	6	6	6	6	6	6	6	-	-	-	-	-	-
d 0 to 7															
Initial BW, g/bird		42.0	41.9	41.4	42.5	41.4	41.9	42.4	41.8	1.0	-	0.077	0.606	-	-
ADG, g/bird/d		17.7	19.9	20.0	18.8	17.8	18.3	19.6	19.8	1.5	-	0.009	0.037	-	-
ADFI, g/bird/d		23.8	25.1	25.2	24.7	23.0	24.0	25.6	24.1	1.0	-	0.006	0.001	-	-
G:F, g/g		0.743	0.795	0.801	0.766	0.775	0.764	0.767	0.819	0.070	-	0.240	0.772	-	-
Final BW, g/bird		165.0	181.6	185.0	174.1	166.0	169.8	179.9	180.4	11.0	-	0.005	0.019	-	-
d 7 to 14															
ADG, g/bird/d		35.0	40.0	39.6	40.6	31.0	40.0	43.5	41.3	3.8	0.875	< 0.001	0.001	0.075	0.115
ADFI, g/bird/d		42.9	47.9	50.2	46.5	39.1	45.7	49.3	47.8	3.6	0.186	< 0.001	< 0.001	0.077	0.862
G:F, g/g		0.816	0.834	0.792	0.872	0.791	0.876	0.880	0.863	0.040	0.044	0.002	0.381	0.369	0.001
Final BW, g/bird		409.8	461.7	462.1	458.0	383.2	450.0	484.1	469.6	30.6	0.893	< 0.001	< 0.001	0.068	0.479
d 14 to 21															
ADG, g/bird/d		65.1	68.1	70.0	71.4	56.2	69.3	62.9	71.8	5.1	0.019	< 0.001	0.326	0.146	0.669
ADFI, g/bird/d		84.4	87.6	88.2	86.8	75.5	83.6	79.9	85.1	4.8	< 0.001	0.012	0.189	0.173	0.773
G:F, g/g		0.772	0.780	0.793	0.822	0.744	0.830	0.788	0.843	0.051	0.530	0.003	0.872	0.479	0.382
Final BW, g/bird		865.5	938.5	952.3	957.6	776.8	934.9	924.4	971.9	42.6	0.037	< 0.001	0.001	0.013	0.387
d 0 to 21															
ADG, g/bird/d		39.3	42.7	43.4	43.6	35.0	42.5	42.0	44.3	2.0	0.037	< 0.001	0.001	0.013	0.402
ADFI, g/bird/d		50.3	53.5	54.5	52.7	45.9	51.1	51.6	52.4	2.2	< 0.001	< 0.001	0.001	0.040	0.820
G:F, g/g		0.780	0.798	0.796	0.828	0.762	0.833	0.815	0.846	0.030	0.116	< 0.001	0.435	0.226	0.129

FTU = phytase unit; G:F = gain-to-feed ratio; HS = heat stress; RMSE = root mean square error; TN = thermoneutral.

1 Temperatures were identical between the TN and HS rooms from d 0 to 7 (28-30°C). From d 7 to 21, the HS room temperature was increased to 34-38°C for 8 h/d (08:00 to 16:00) and then reduced to 26-29°C from 16:00 to 08:00 the following day, representing a cyclic heat stress pattern that was maintained until the end of the study. The TN room temperature was maintained at 26-29°C from d 7 to 21.

2 Linear (L) and quadratic (Q) effects represented the responses to increasing dietary phytase levels.

For bone mineralization variables, birds fed the PC diet showed greater (*P* < 0.05) fat-free bone weight (g), bone ash weight (g), percent bone ash (fat-free basis), and bone breaking strength in both the tibia and femur compared with birds fed the NC diet (Table 4). Femur breaking strength was greater (*P* = 0.039) in birds reared under TN than in those reared under HS. Increasing phytase supplementation linearly increased (*P* = 0.017) fat-free tibia weight (g), whereas fat-free femur weight exhibited both linear and quadratic responses (*P* < 0.05; Table 5). Bone ash weight (g), bone ash percentage (fat-free basis), and bone breaking strength increased (*P* ≤ 0.001) linearly and quadratically with increasing phytase supplementation.

**Table 4:** Effects of temperature (T) and reduced dietary calcium and phosphorus concentrations on bone mineralization in broilers.

Item	T^1^:	TN		HS		SEM	*P*-values		
	Diet^2^:	PC	NC	PC	NC		T	Diet	T × Diet
*n*		6	6	6	6	-	-	-	-
Tibia									
Fat-free tibia weight, g		4.63	4.03	4.35	3.53	0.28	0.179	0.021	0.702
Fat-free tibia weight, % BW		0.48	0.46	0.46	0.46	0.03	0.573	0.740	0.761
Tibia ash weight, g		2.49	1.63	2.37	1.58	0.08	0.324	< 0.001	0.669
Tibia ash weight, % fat-free tibia weight		53.63	42.61	54.45	44.94	1.97	0.433	< 0.001	0.705
Tibia breaking strength, N		234.1	126.9	231.2	118.1	10.8	0.594	< 0.001	0.788
Femur									
Fat-free femur weight, g		3.42	2.49	3.19	2.58	0.14	0.632	< 0.001	0.257
Fat-free femur weight, % BW		0.36	0.29	0.33	0.33	0.02	0.470	0.062	0.061
Femur ash weight, g		1.77	1.06	1.67	1.06	0.06	0.407	< 0.001	0.452
Femur ash weight, % fat-free femur weight		51.95	42.87	52.43	41.22	0.63	0.364	< 0.001	0.106
Femur breaking strength, N		196.7	65.2	171.2	49.2	9.1	0.039	< 0.001	0.624

HS = heat stress; NC = negative control; PC = positive control; TN = thermoneutral.

1 Temperatures were identical between the TN and HS rooms from d 0 to 7 (28-30°C). From d 7 to 21, the HS room temperature was increased to 34-38°C for 8 h/d (08:00 to 16:00) and then reduced to 26-29°C from 16:00 to 08:00 the following day, representing a cyclic heat stress pattern that was maintained until the end of the study. The TN room temperature was maintained at 26-29°C from d 7 to 21.

2 The PC diet contained 100% of the recommended calcium and phosphorus levels, whereas the NC diet contained 83% and 71% of the recommended levels for calcium and phosphorus, respectively.

**Table 5:** Effects of temperature (T) and phytase supplementation levels on bone mineralization in broilers^1^.

Item	T:	TN				HS				RMSE	*P*-values for contrasts^2^				
	Phytase (FTU/kg):	0	500	1,000	1,500	0	500	1,000	1,500		T	L	Q	T × L	T × Q
*n*		6	6	6	6	6	6	6	6	-	-	-	-	-	-
Tibia															
Fat-free tibia weight, g		4.03	4.40	4.42	4.21	3.53	4.13	4.22	4.41	0.52	0.202	0.017	0.106	0.107	0.778
Fat-free tibia weight, % BW		0.46	0.47	0.46	0.44	0.46	0.44	0.46	0.45	0.05	0.658	0.634	0.779	0.533	0.510
Tibia ash weight, g		1.63	2.26	2.33	2.23	1.58	2.13	2.25	2.31	0.18	0.378	< 0.001	< 0.001	0.348	0.243
Tibia ash weight, % fat-free tibia weight		42.61	51.47	52.60	53.03	44.94	51.42	53.29	52.35	3.64	0.590	< 0.001	0.001	0.382	0.810
Tibia breaking strength, N		126.9	196.3	224.9	222.3	118.1	197.4	208.8	217.1	29.3	0.397	< 0.001	< 0.001	0.936	0.976
Femur															
Fat-free femur weight, g		2.49	3.10	3.17	3.11	2.58	2.83	3.06	3.16	0.30	0.474	< 0.001	0.022	0.978	0.130
Fat-free femur weight, % BW		0.29	0.33	0.33	0.33	0.33	0.30	0.33	0.33	0.03	0.683	0.206	0.534	0.196	0.056
Femur ash weight, g		1.06	1.49	1.60	1.58	1.06	1.39	1.57	1.62	0.14	0.594	< 0.001	< 0.001	0.567	0.336
Femur ash weight, % fat-free femur weight		42.87	48.16	50.28	50.68	41.22	49.34	51.32	51.18	1.65	0.575	< 0.001	< 0.001	0.145	0.085
Femur breaking strength, N^3^		65.2	135.0	172.8	151.3	49.2	135.0	166.9	154.9	26.4	0.553	< 0.001	< 0.001	0.451	0.834

FTU = phytase unit; HS = heat stress; RMSE = root mean square error; TN = thermoneutral.

1 Temperatures were identical between the TN and HS rooms from d 0 to 7 (28-30°C). From d 7 to 21, the HS room temperature was increased to 34-38°C for 8 h/d (08:00 to 16:00) and then reduced to 26-29°C from 16:00 to 08:00 the following day, representing a cyclic heat stress pattern that was maintained until the end of the study. The TN room temperature was maintained at 26-29°C from d 7 to 21.

2 Linear (L) and quadratic (Q) effects represented the responses to increasing dietary phytase levels.

3 The TN treatment with 0 FTU/kg phytase had 5 replicates.

Regarding nutrient digestibility and utilization, birds fed the NC diet exhibited greater (*P* < 0.001) AID of Ca and greater (*P* = 0.001) P utilization compared with birds fed the PC diet (Table 6). Heat stress also increased (*P* < 0.05) both AID and utilization of P compared with TN. Interactions between temperature and diet were observed for AID of GE, AID of N, GE utilization, DM utilization, AME (as-fed and DM basis), and N retention (*P* < 0.05). Under TN conditions, reducing Ca and P concentrations from PC to NC diets did not affect these variables, whereas under HS conditions the PC diet resulted in greater values than the NC diet. For N utilization, values were greater under HS than TN in birds fed the PC diet, whereas dietary treatment did not affect N utilization in birds fed the NC diet (*P* = 0.001). An interaction between temperature and diet was observed for N intake (*P* = 0.049), such that temperature did not affect N intake in birds fed the PC diet, whereas birds fed the NC diet had greater N intake under TN than under HS. An interaction between temperature and diet was also observed for N excretion (*P* = 0.022), such that temperature did not affect N excretion in birds fed the NC diet, whereas birds fed the PC diet had greater N excretion under TN than under HS.

**Table 6:** Effects of temperature (T) and reduced dietary calcium (Ca) and phosphorus (P) concentrations on energy and nutrients digestibility and utilization and nitrogen (N) balance in broilers.

Item	T^1^:	TN		HS		SEM	*P*-values		
	Diet^2^:	PC	NC	PC	NC		T	Diet	T × Diet
*n*		6	6	6	6	-	-	-	-
Apparent ileal digestibility									
GE, %		73.0^ab^	72.3^b^	75.0^a^	70.9^b^	0.6	0.656	0.001	0.015
N, %		84.3^a^	83.4^a^	84.4^a^	81.9^b^	0.4	0.074	< 0.001	0.047
Ca, %		60.5	68.7	59.9	72.3	1.6	0.337	< 0.001	0.192
P, %		66.6	66.3	68.6	71.2	1.0	0.003	0.270	0.166
Apparent utilization									
GE, %		73.1^ab^	73.3^ab^	75.6^a^	72.9^b^	0.6	0.131	0.061	0.032
DM, %		68.3^b^	68.8^ab^	70.7^a^	68.2^b^	0.6	0.176	0.159	0.030
N, %		62.4^b^	66.6^a^	67.9^a^	64.6^ab^	0.9	0.077	0.655	0.001
Ca, %		57.0	54.7	60.4	59.7	2.1	0.061	0.485	0.721
P, %		52.4	60.5	57.2	65.6	2.0	0.026	0.001	0.933
AME, kcal/kg as-fed		3,013^b^	3,035^b^	3,113^a^	3,016^b^	26	0.133	0.156	0.032
AMEn, kcal/kg as-fed		2,830	2,840	2,913	2,827	24	0.154	0.125	0.055
AME, kcal/kg DM		3,330^b^	3,353^b^	3,440^a^	3,332^b^	28	0.133	0.156	0.032
AMEn, kcal/kg DM		3,127	3,138	3,219	3,124	26	0.154	0.125	0.055
N balance									
N intake, g/bird/d		1.94^a^	1.79^b^	1.90^ab^	1.63^c^	0.03	0.002	< 0.001	0.049
N retention, g/bird/d		1.21^a^	1.19^a^	1.29^a^	1.05^b^	0.03	0.254	< 0.001	< 0.001
N excretion, g/bird/d		0.73^a^	0.60^b^	0.61^b^	0.58^b^	0.02	0.002	< 0.001	0.022

GE = gross energy; HS = heat stress; NC = negative control; PC = positive control; TN = thermoneutral.

^a-b^Means within a row without a common superscript letter differ (*P* < 0.05) based on the interaction.

1 Temperatures were identical between the TN and HS rooms from d 0 to 7 (28-30°C). From d 7 to 21, the HS room temperature was increased to 34-38°C for 8 h/d (08:00 to 16:00) and then reduced to 26-29°C from 16:00 to 08:00 the following day, representing a cyclic heat stress pattern that was maintained until the end of the study. The TN room temperature was maintained at 26-29°C from d 7 to 21.

2 The PC diet contained 100% of the recommended calcium and phosphorus levels, whereas the NC diet contained 83% and 71% of the recommended levels for calcium and phosphorus, respectively.

Apparent ileal digestibility and apparent utilization of P were greater under HS than under TN (*P* < 0.001), whereas N excretion was greater under TN than under HS (*P* = 0.001; Table 7). Increasing phytase supplementation decreased AID of Ca linearly and quadratically (*P* < 0.05) but increased Ca utilization linearly and quadratically (*P* < 0.05). Phytase supplementation also linearly increased (*P* < 0.05) AID of GE, AID of N, AID of P, GE utilization, DM utilization, AME, and AMEn, whereas P utilization and N excretion increased (*P* < 0.05) both linearly and quadratically. In addition, interactions between temperature and the linear effect of phytase supplementation were observed for N intake and N retention (*P* < 0.05), such that both variables increased linearly with increasing phytase levels under TN and HS conditions, but the rate of increase was greater under HS, being approximately 6.7-fold higher for N intake and 2.5-fold higher for N retention than under TN.

**Table 7:** Effects of temperature (T) and phytase supplementation levels on energy and nutrients digestibility and utilization and nitrogen (N) balance in broilers^1^.

Item	T:	TN				HS				RMSE	*P*-values for contrasts^2^				
	Phytase (FTU/kg):	0	500	1,000	1,500	0	500	1,000	1,500		T	L	Q	T × L	T × Q
*n*		6	6	6	6	6	6	6	6	-	-	-	-	-	-
Apparent ileal digestibility															
GE, %		72.3	73.0	73.8	75.0	70.9	72.2	73.7	74.4	2.0	0.228	< 0.001	0.987	0.537	0.634
N, %		83.4	84.2	84.3	86.0	81.9	83.5	84.6	85.0	1.4	0.077	< 0.001	0.836	0.506	0.202
Ca, %		68.7	65.1	61.6	63.0	72.3	63.8	61.9	63.5	5.7	0.635	0.002	0.028	0.595	0.436
P, %		66.3	68.0	72.4	76.1	71.2	73.5	74.9	80.8	3.4	< 0.001	< 0.001	0.152	0.684	0.684
Apparent utilization															
GE, %		73.3	73.7	73.9	74.1	72.9	73.7	74.0	75.0	1.6	0.781	0.035	0.954	0.324	0.859
DM, %		68.8	69.4	69.3	69.9	68.2	69.1	69.4	70.6	1.7	0.970	0.017	0.861	0.331	0.896
N, %		66.6	63.1	63.5	64.6	64.6	66.1	65.2	66.9	2.5	0.086	0.970	0.110	0.086	0.132
Ca, %		54.7	65.9	63.9	63.7	59.7	61.8	68.4	67.0	4.0	0.066	< 0.001	0.003	0.699	0.093
P, %		60.5	72.3	72.8	73.4	65.6	70.9	76.5	75.6	2.4	0.001	< 0.001	< 0.001	0.577	0.084
AME, kcal/kg as-fed		3,035	3,048	3,057	3,066	3,016	3,048	3,061	3,102	67	0.781	0.035	0.954	0.324	0.859
AMEn, kcal/kg as-fed		2,840	2,864	2,871	2,877	2,827	2,855	2,871	2,907	61	0.925	0.021	0.895	0.382	0.705
AME, kcal/kg DM		3,353	3,368	3,378	3,388	3,332	3,368	3,383	3,428	74	0.781	0.035	0.954	0.324	0.859
AMEn, kcal/kg DM		3,138	3,165	3,173	3,179	3,124	3,154	3,172	3,212	67	0.925	0.021	0.895	0.382	0.705
N balance															
N intake, g/bird/d		1.79	1.90	1.94	1.87	1.63	1.82	1.83	1.86	0.08	< 0.001	< 0.001	0.001	0.040	0.820
N retention, g/bird/d		1.19	1.20	1.23	1.21	1.05	1.20	1.20	1.24	0.07	0.116	0.001	0.129	0.015	0.431
N excretion, g/bird/d		0.60	0.70	0.71	0.66	0.58	0.62	0.64	0.62	0.05	0.001	0.012	0.001	0.709	0.139

Ca = calcium; FTU = phytase unit; GE = gross energy; HS = heat stress; P = phosphorus; RMSE = root mean square error; TN = thermoneutral.

1 Temperatures were identical between the TN and HS rooms from d 0 to 7 (28-30°C). From d 7 to 21, the HS room temperature was increased to 34-38°C for 8 h/d (08:00 to 16:00) and then reduced to 26-29°C from 16:00 to 08:00 the following day, representing a cyclic heat stress pattern that was maintained until the end of the study. The TN room temperature was maintained at 26-29°C from d 7 to 21.

2 Linear (L) and quadratic (Q) effects represented the responses to increasing dietary phytase levels.

**Figure 2:**
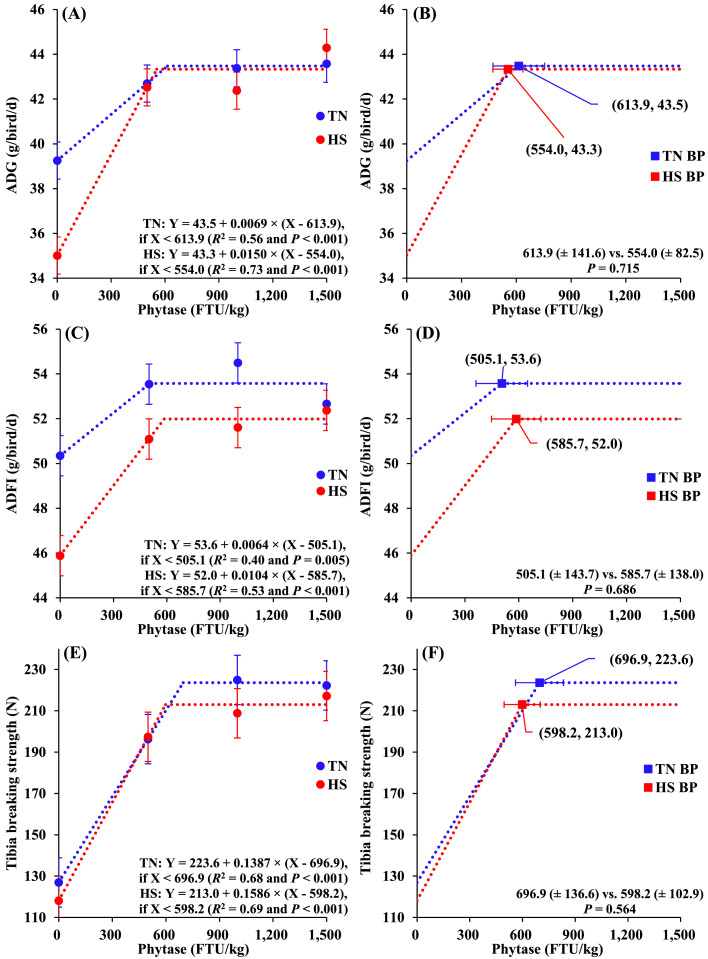

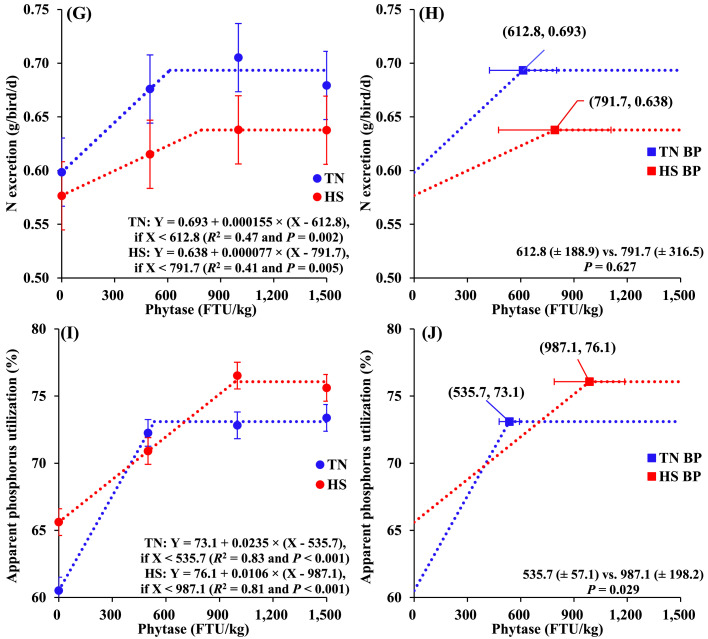
Estimation and comparison of optimal dietary phytase levels between thermoneutral (TN) and heat stress (HS) conditions using one-slope broken-line regression analysis. Broilers were fed diets containing 0, 500, 1,000, or 1,500 phytase units (FTU) per kg of diet, and responses were evaluated for ADG (d 0 to 21; Figures A and B), ADFI (d 0 to 21; Figures C and D), tibia breaking strength (Figures E and F), nitrogen (N) excretion (Figures G and H), and apparent phosphorus utilization (Figures I and J). Figures A, C, E, G, and I present least squares means ± SEM across phytase levels for TN and HS conditions, fitted with one-slope broken-line regression models. Corresponding *R*^2^ values and model *P*-values are shown within each panel. Figures B, D, F, H, and J display the estimated break-points (BP), representing the optimal dietary phytase level for each thermal condition, along with the statistical comparison of BP estimates between TN and HS within the same response variable.

Under TN conditions, the breakpoint for ADG was estimated at 613.9 FTU/kg (*R*^2^ = 0.56, *P* < 0.001), whereas under HS conditions it was 554.0 FTU/kg (*R*^2^ = 0.73, *P* < 0.001; Figure 2**A**). The estimated optimal phytase level for ADG did not differ between environmental conditions (*P* = 0.715; Figure 2**B**). For ADFI, breakpoints were estimated at 505.1 FTU/kg under TN (*R*^2^ = 0.40, *P* = 0.005) and 585.7 FTU/kg under HS (*R*^2^ = 0.53, *P* < 0.001; Figure 2**C**), with no difference between rearing temperatures (*P* = 0.686; Figure 2**D**). For tibia breaking strength, breakpoints were 696.9 FTU/kg under TN (*R*^2^ = 0.68, *P* < 0.001) and 598.2 FTU/kg under HS (*R*^2^ = 0.69, *P* < 0.001; Figure 2**E**), and the estimated optimal phytase levels did not differ between TN and HS (*P* = 0.564; Figure 2**F**). For N excretion, breakpoints were estimated at 612.8 FTU/kg under TN (*R*^2^ = 0.47, *P* = 0.002) and 791.7 FTU/kg under HS (*R*^2^ = 0.41, *P* = 0.005; Figure 2**G**), with no difference between conditions (*P* = 0.627; Figure 2**H**). For apparent utilization of P, the breakpoint was estimated at 535.7 FTU/kg under TN (*R*^2^ = 0.83, *P* < 0.001) and 987.1 FTU/kg under HS (*R*^2^ = 0.81, *P* < 0.001; Figure 2**I**). The estimated optimal phytase level was greater under HS than under TN (*P* = 0.029; Figure 2**J**).

Least squares means of ADG, ADFI, tibia breaking strength, and N excretion in broilers fed the PC diet did not differ from the estimated plateau responses obtained from one-slope broken-line analysis under either TN or HS conditions (Table 8). In contrast, supplementing the NC diet with the estimated optimal phytase levels of 535.7 and 987.1 FTU/kg under TN and HS conditions, respectively, resulted in greater (*P* < 0.001) P utilization than that of birds fed the PC diet.

**Table 8:** Comparison of least squares means (LSM) of responses in broilers fed the positive control (PC) diet with estimated plateau responses from one-slope broken-line analysis of graded phytase supplementation to the negative control (NC) diet under thermoneutral (TN) and heat stress (HS) conditions.

Item	PC diet^1^		NC diet supplemented with the optimal phytase level^2^			*P*-value^3^
	LSM	SE	Plateau	SE	Optimal phytase level (FTU/kg)	
ADG, g/bird/d^4^						
TN	43.7	1.0	43.5	0.5	613.9	0.820
HS	43.3	0.8	43.3	0.7	554.0	1.000
ADFI, g/bird/d^4^						
TN	54.2	1.1	53.6	0.5	505.1	0.624
HS	53.0	0.5	52.0	0.7	585.7	0.261
Tibia breaking strength, N						
TN	234.1	8.9	223.6	8.3	696.9	0.389
HS	231.2	17.5	213.0	8.2	598.2	0.347
Nitrogen excretion, g/bird/d						
TN	0.728	0.026	0.693	0.014	612.8	0.234
HS	0.608	0.015	0.638	0.010	791.7	0.109
Apparent utilization of P, %						
TN	52.4	3.4	73.1	0.7	535.7	< 0.001
HS	57.2	1.8	76.1	0.6	987.1	< 0.001

FTU = phytase unit; P = phosphorus.

1 Values represent LSM and their SE for broilers fed the PC diet (*n* = 6).

2 Values represent the estimated plateau response, its SE, and the corresponding optimal phytase level obtained from one-slope broken-line analysis of responses to graded phytase supplementation in broilers fed the NC diet.

3 *P*-values were obtained using a two-sample z-test comparing the LSM with the estimated plateau response within each thermal condition.

4 Values for ADG and ADFI were measured from d 0 to 21.

## Discussion

Phytase activity analyzed immediately after mixing at the feed mill showed a strong linear relationship with calculated values, with a slope close to unity, indicating uniform and accurate incorporation of phytase into the experimental diets (Figure 1). After 21 d of storage, phytase activity decreased by 6.3% under TN and by 17.2% under HS relative to d 0, regardless of supplementation level. This reduction over time is consistent with previous reports (Naves et al., 2012; De Jong et al., 2016, 2017) and was likely associated with environmental effects on enzyme stability, particularly elevated temperature, which can promote denaturation and conformational changes that reduce enzyme activity (Ward, 2002; Sulabo et al., 2011). The greater loss under HS suggests that elevated temperature accelerated phytase degradation, as also reported by Sulabo et al. (2011) and De Jong et al. (2016). However, diets fed to birds in the present study were not stored under HS conditions; instead, all diets were stored under TN conditions and provided to feeders daily. Therefore, given the 21-d experimental period and the relative stability of phytase under room temperature or lower storage conditions reported by Sulabo et al. (2011), calculated phytase inclusion levels were considered appropriate for statistical analysis and estimation of optimal supplementation levels.

Because HS was not imposed until d 7, only diet was included as a fixed effect in the statistical model for growth performance during d 0 to 7 (Tables 2 **and** 3). Consistent with this approach, when temperature, diet, and their interaction were included in the model for this pre-challenge period, neither temperature nor the temperature × diet interaction affected growth performance (data not shown).

From d 7 onward, birds were subjected to cyclic HS (Alabi et al., 2026). Because one of the major objectives of this study was to estimate the optimal supplemental level of phytase under commercially relevant conditions, the cyclic HS model was considered more appropriate than a continuous HS model. Among HS models, cyclic HS may better reflect commercial production environments because birds are more likely to experience diurnal fluctuations in ambient temperature (Andretta et al., 2021). Therefore, the present findings should be interpreted specifically within the context of cyclic HS and should not be directly extrapolated to continuous HS conditions, as bird responses may differ depending on the pattern of heat exposure. Compared with TN conditions, previous studies have shown that cyclic HS (8 h/d) reduces ADG (Zhang et al., 2012; de Souza et al., 2016; Teyssier et al., 2022) and ADFI (de Souza et al., 2016; Teyssier et al., 2022), although the magnitude of these reductions is significantly less than that observed under continuous HS (24 h/d). In contrast, feed efficiency appears to be more consistently impaired under continuous HS, whereas cyclic HS often has little or no effect on G:F or FCR relative to TN conditions (de Souza et al., 2016; Teyssier et al., 2022). This pattern suggests that, although cyclic HS negatively affected growth performance in the present study, its impact was likely moderate compared with that expected under continuous HS. It also indicates that the reduction in ADG under cyclic HS was driven primarily by reduced feed intake rather than by a substantial decline in feed efficiency, and that a more severe heat challenge may be required before G:F is markedly impaired.

Although HS had little effect on bone mineralization in the present study (Tables 4 **and** 5), this may be related to the relatively short duration of HS exposure. In this experiment, birds were exposed to HS only from d 7 to 21, representing a 14-d challenge period, which may not have been long enough for clear detrimental effects on bone mineralization to fully develop. Señas-Cuesta et al. (2023) reported no difference between TN and cyclic HS (35°C for 12 h/d) conditions in tibia breaking strength or tibia ash concentration at d 10, whereas these variables were greater under TN conditions at d 42. Similarly, Rocchi et al. (2022) observed no difference in tibia breaking strength or tibia ash concentration between TN and HS conditions at d 21, but greater values under TN conditions at d 42. Although these previous studies did not always detect statistically significant differences at the earlier time points, responses under HS were numerically lower than those under TN conditions. A similar pattern was observed in the present study, where several bone-related responses, such as bone ash and bone breaking strength, were numerically lower under HS despite the lack of statistical significance. Because the 14-d HS period used in the present study falls between the intermediate sampling times reported in those previous studies (Rocchi et al., 2022; Señas-Cuesta et al., 2023), it is possible that the duration of HS was insufficient to allow significant differences in bone mineralization to be detected. Therefore, a longer period of HS exposure may have resulted in clearer negative effects on bone mineralization.

In the present study, broilers reared under HS exhibited greater AID and utilization of P than birds kept under TN conditions (Tables 6 **and** 7). This response should not be interpreted as an improvement in physiological status, because HS is generally associated with impaired intestinal integrity, altered nutrient transporter expression, endocrine disruption, and reduced bone mineralization (Teyssier et al., 2023). A more plausible explanation is a compensatory adjustment in mineral metabolism under HS. Under thermal challenge, reduced feed intake and shifts in metabolic demand may effectively reduce dietary P intake relative to physiological requirements, thereby activating regulatory mechanisms that enhance the efficiency of P acquisition and retention (Teyssier et al., 2023). This interpretation is supported by previous studies demonstrating that birds subjected to P limitation can maintain mineral homeostasis through coordinated endocrine and intestinal adaptations to maintain P homeostasis. Specifically, reduced P supply has been shown to stimulate hormonal responses that increase renal P conservation while simultaneously enhancing the production of active 1,25-dihydroxycholecalciferol, which in turn promote intestinal P absorption and mobilization of mineral reserves from bone (Omotoso et al., 2023). In parallel, low dietary P has been associated with an upregulation of transcellular P transport capacity in the intestine, likely through increased expression of sodium-dependent phosphate transporters such as NaPi-IIb, thereby improving the efficiency of P uptake (Liu et al., 2017). Furthermore, prior exposure to reduced non-phytate P supply has been reported to enhance subsequent P utilization, including greater digestibility and increased phytate degradation, suggesting that early nutritional conditions can induce adaptive changes that persist later in life (Haetinger et al., 2024). Nevertheless, because the present estimates were based on apparent rather than standardized digestibility, the potential contribution of HS-induced changes in endogenous P losses cannot be ruled out (Babatunde et al., 2020).

The NC diet was formulated with reduced Ca and non-phytate P concentrations relative to the PC diet to allow evaluation of phytase efficacy under a marginal Ca and P deficiency. The magnitude of the reduction was determined using the nutrient matrix values. Thus, Ca and non-phytate P were reduced by 17% and 45%, respectively, in the NC diet compared with the PC diet. At the highest phytase inclusion level used in the present study, 1,500 FTU/kg, the expected Ca and non-phytate P release based on the matrix values was intended to compensate for these reductions, resulting in an available Ca and non-phytate P supply comparable to that of the PC diet. This approach enabled the assessment of phytase dose responses while maintaining the PC diet as the reference treatment for adequate mineral nutrition. Reducing dietary Ca and P from the PC to the NC impaired overall growth performance and markedly reduced bone mineralization (Tables 2 **and** 4). These findings indicate that the mineral reduction applied in the NC diet was sufficient to limit not only skeletal mineral deposition, but also productive performance. This is consistent with previous work showing that inadequate P supply in young broilers depresses growth and skeletal development because Ca and P become limiting for hydroxyapatite deposition and because absorbed minerals are preferentially directed toward homeostatic maintenance rather than productive growth (Proszkowiec-Weglarz and Angel, 2013; Liu et al., 2017).

An important finding was the diet and temperature interaction observed for ADFI from d 0 to 21 (*P* = 0.048), with a similar tendency for ADG (*P* = 0.073; Table 2). In birds fed the PC diet, ADFI and ADG were relatively similar between TN and HS conditions, whereas in birds fed the NC diet, both responses were lower under HS than under TN. This suggests that, under a cyclic HS challenge, adequate Ca and P supply may have helped buffer the negative effects of HS on feed intake and growth, whereas this buffering capacity was diminished when birds were fed the mineral-reduced NC diet. This interpretation is further supported by nutrient digestibility and utilization responses (Table 6). Under TN conditions, PC and NC diets produced similar AID of GE, AID of N, GE utilization, DM utilization, AME, and N retention; however, under HS conditions, these responses were greater in birds fed the PC diet than in those fed the NC diet. Thus, the poorer performance of birds fed the NC diet under HS appears to have resulted from the combined effects of lower feed intake and reduced digestive and postabsorptive nutrient use. This agrees with previous reports that HS can impair nutrient digestibility and alter intestinal and metabolic function in broilers (de Souza et al., 2016; Teyssier et al., 2023).

However, birds fed the NC diet showed greater AID of Ca and P utilization than birds fed the PC diet (Table 6). This outcome was unexpected because the PC and NC diets contained the same calculated phytate P concentration, whereas the PC diet supplied more non-phytate P. Because broilers utilize phytate-bound P poorly owing to limited endogenous phytase activity (Akter et al., 2016; Haetinger et al., 2024), and because non-phytate P is more available than phytate P, greater P utilization would ordinarily have been expected in birds fed the PC diet. In agreement with that expectation, Hamdi et al. (2015) reported that when phytate P was held constant at 0.25%, increasing dietary non-phytate P from 0.30 to 0.45% increased AID of P. Furthermore, the P utilization in the present study was expressed as apparent rather than standardized or true estimates. Apparent values do not correct for basal endogenous P losses (Kong and Adeola, 2014), and those losses can exert a proportionally greater influence on apparent coefficients when dietary P concentration is low (Stein et al., 2007). A more plausible explanation, therefore, is that birds fed the more P-adequate PC diet excreted more P because absorbed P more readily exceeded physiological demand, whereas birds fed the mineral-restricted NC diet conserved P more efficiently. This is also consistent with evidence that low-P feeding stimulates endocrine and intestinal adaptations involved in P acquisition and conservation, and that prior exposure to reduced dietary non-phytate P can increase subsequent P digestibility in broilers (Proszkowiec-Weglarz and Angel, 2013; Omotoso et al., 2023; Haetinger et al., 2024). The concomitant increase in AID of Ca may reflect the same deficiency-driven homeostatic adjustment. This interpretation is further supported by the graded phytase responses observed in the present study. As phytase supplementation increased in the NC diet, AID of P increased linearly (*P* < 0.001) without a quadratic response (*P* = 0.152), whereas P utilization increased quadratically (*P* < 0.001) and eventually reached a plateau (*P* < 0.001). This pattern suggests that phytase progressively increased ileal P disappearance, but the additional absorbed P was not retained proportionally once the bird’s physiological requirement was approached or exceeded. In other words, beyond a certain point, additional P released by phytase may have been excreted rather than retained, resulting in a plateau in P utilization despite the continued linear increase in AID of P.

Before the thermal challenge began, phytase supplementation linearly and quadratically increased ADG and ADFI from d 0 to 7, indicating that the NC diet was sufficiently deficient in available minerals (Table 3). Over the overall experimental period, phytase linearly increased G:F, and marked improvements were observed in bone responses (Table 5), supporting the interpretation that phytase released phytate-bound P and improved mineral availability for skeletal development. These responses are consistent with the established role of exogenous phytase in hydrolyzing phytate, increasing P availability, and reducing the antinutritive effects of phytate in poultry diets (Adeola et al., 2004; Lei et al., 2013; Adejumo et al., 2021). Phytase supplementation also improved AID of GE, N, and P, as well as apparent utilization of GE, DM, Ca, and P, together with AME and AMEn (Table 7). These responses indicate that the benefits of phytase extended beyond P release and likely included reduced phytate interference with the utilization of other nutrients (Gehring et al., 2013; Walk et al., 2013). By hydrolyzing phytate, phytase can decrease mineral-phytate complex formation and improve access to nutrients that would otherwise remain partially unavailable in the gastrointestinal tract (Lei et al., 2013). The increase in P utilization was especially important because it indicates that P released from phytate was not only digested but also retained and utilized by the bird.

One response that requires careful interpretation is the decrease in AID of Ca with increasing phytase supplementation, despite the concurrent increase in Ca utilization. This difference may be related to the site of measurement and physiological regulation of mineral utilization. Apparent ileal digestibility reflects mineral disappearance up to the ileum, whereas apparent utilization reflects whole-tract utilization after post-ileal processes and excretion. In the present study, the unsupplemented NC diet likely stimulated compensatory Ca absorption at the ileal level because of mineral deficiency, whereas phytase supplementation improved overall Ca balance and reduced the need for this compensatory response. Therefore, the decrease in AID of Ca should not be interpreted as impaired Ca nutrition, particularly because Ca utilization, bone mineralization, and growth performance were improved. This broader response pattern suggests that phytase improved overall Ca utilization despite lowering apparent ileal Ca disappearance. Absolute N excretion also increased with phytase supplementation. However, because N excretion was expressed as g/bird/d, this increase most likely reflected greater feed and N intake in phytase-supplemented birds rather than poorer N-use efficiency. This is supported by the concurrent increase in N retention and the lack of a clear negative response in N utilization.

A temperature × phytase interaction was observed for the linear responses of ADG, ADFI, and N retention, such that these variables increased linearly with increasing phytase supplementation under both TN and HS conditions, but the rate of increase was greater under HS than under TN, being 2.5-fold higher for ADG, 2.0-fold higher for ADFI, and 2.5-fold higher for N retention. This suggests that birds exposed to HS obtained a greater marginal benefit from each increment of phytase supplementation. Because HS reduced feed intake and can impair digestive and metabolic function, improving nutrient release from each unit of feed may have been more important under HS than under TN conditions (de Souza et al., 2016; Teyssier et al., 2023). This interpretation is consistent with Lee et al. (2019), who reported that phytase improved weight gain and feed intake in broilers exposed to high environmental temperatures. In contrast, the absence of temperature and phytase interactions for most bone and P-utilization responses suggests that the fundamental efficacy of phytase was maintained under both thermal environments, even though its effects on growth and N retention were more pronounced under HS.

Broken-line analysis showed that the estimated optimal phytase level depended on the response criterion (Figure 2). For ADG, ADFI, tibia breaking strength, and N excretion, the estimated optimal levels ranged from approximately 500 to 800 FTU/kg and did not differ between TN and HS conditions. These results indicate that 800 FTU/kg phytase supplementation was sufficient to maximize growth performance and bone strength in broilers fed the NC diet under both thermal environments. Beyond this level, additional phytase supplementation appeared to provide limited marginal improvement in these responses, likely because the dose-response relationship approached a plateau, reflecting diminishing returns for growth performance and bone strength (Sung and Adeola, 2023). In contrast, the estimated optimal phytase level for P utilization differed between thermal environments. This was the only response criterion for which the estimated optimum was greater under HS than under TN. A likely explanation is that HS reduced feed intake and altered mineral metabolism, thereby increasing the importance of phytase-mediated P release and retention from each unit of feed consumed. This interpretation is consistent with the concept that birds under limited P supply can activate adaptive mechanisms to enhance P acquisition and conservation (Proszkowiec-Weglarz and Angel, 2013; Omotoso et al., 2023). Thus, the optimal phytase level should not be viewed as a single fixed value, but rather as a response-specific estimate that depends on the biological endpoint being optimized.

It is also important to note that the optimal phytase levels estimated in the present study were not likely overestimated because of heat-induced loss of phytase activity in the feed. Although the phytase stability assay showed greater loss of analyzed activity under HS conditions, the diets actually consumed by the birds were stored under TN conditions and supplied to feeders daily. Therefore, the greater optimal phytase level for P utilization under HS likely reflects a biological response of the birds to thermal challenge rather than reduced phytase recovery in the diet.

The comparison between the broken-line plateau responses and the LSM responses of birds fed the PC diet (Table 8) indicated that phytase supplementation at approximately 700 FTU/kg under TN and 1,000 FTU/kg under HS would restore most key responses of broilers fed the reduced Ca and P diet to values comparable with those of birds fed the PC diet. Interestingly, the plateau response for P utilization exceeded the LSM of birds fed the PC diet under both thermal environments. This suggests that, when phytase was supplemented at the level required to maximize P utilization, birds fed the NC diet utilized P more efficiently than those fed the conventional PC formulation. Therefore, if the primary objective is to maximize P utilization and reduce P excretion, selecting the phytase dose based only on growth performance may underestimate the level needed, particularly under HS conditions.

Finally, caution is needed when applying the optimal phytase levels estimated in the present study. These values may vary depending on the response criterion evaluated, thermal environment, feed storage temperature, bird age, dietary phytate-P concentration, and duration of feed storage. In addition, the optimal levels reported in this study should be interpreted within the context of the statistical model used. Different models, such as quadratic broken-line or quadratic polynomial regression, can generate different estimates from the same dose-response dataset, and quadratic-based models always produce greater estimated optimal levels than linear broken-line models (Vedenov and Pesti, 2008; Pesti et al., 2009). The one-slope broken-line model was used in the present study to provide a consistent basis for comparing response criteria and thermal environments. Therefore, direct comparison of these optimal phytase levels with values from other studies should consider differences in model selection, response criteria, diet composition, environmental conditions, and feed storage practices.

## Conclusions

In conclusion, the phytase stability evaluation showed that analyzed phytase activity declined during feed storage, with greater loss under HS than under TN conditions, confirming that elevated storage temperature can accelerate phytase degradation. This finding should be considered separately from the biological responses of broilers, because the diets actually consumed by birds were stored under TN conditions and supplied to feeders daily. Cyclic HS reduced broiler growth performance, whereas lowering dietary Ca and P impaired growth, markedly reduced bone mineralization, and altered mineral utilization patterns. Phytase supplementation quadratically improved growth performance, bone mineralization, and N excretion in broilers fed the NC diet under both TN and HS conditions. Optimal phytase levels for growth performance, tibia breaking strength, and N excretion were not different between TN and HS conditions. Phosphorus digestibility increased linearly with phytase supplementation, whereas P utilization showed a quadratic response and reached a plateau, with a greater estimated optimal phytase level under HS than under TN. Supplementation of the NC diet with the estimated optimal phytase level restored ADG, ADFI, tibia breaking strength, and N excretion to values comparable with those of the PC diet, while P utilization exceeded that of the PC diet under both thermal environments. Collectively, under the conditions of this 21-d experiment, phytase can effectively compensate for moderate dietary Ca and P reductions in broilers under both TN and cyclic HS conditions, but the phytase level considered optimal depends on both the response criterion and rearing temperature.

## CRediT authorship contribution statement

**Hansol Kim:** Writing – original draft, Visualization, Formal analysis, Data curation. **Sunday A. Adedokun:** Data curation, Writing – review & editing, Supervision, Resources, Project administration, Methodology, Funding acquisition.

## Disclosures

The authors declare that they have no known competing financial interests or personal relationships that could have appeared to influence the work reported in this paper.
